# Perceptual effects of fast and automatic visual ensemble statistics from faces in individuals with typical development and autism spectrum conditions

**DOI:** 10.1038/s41598-020-58971-y

**Published:** 2020-02-07

**Authors:** Mrinmoy Chakrabarty, Makoto Wada

**Affiliations:** 1 Developmental Disorders Section, Department of Rehabilitation for Brain Functions, Research Institute of National Rehabilitation Center for Persons with Disabilities, Tokorozawa, 359-8555 Japan; 2 Department of Social Sciences and Humanities, Indraprastha Institute of Information Technology-Delhi (IIIT-D), Okhla Industrial Estate,Phase III, New Delhi, 110020 India

**Keywords:** Perception, Human behaviour

## Abstract

We investigated whether covert ensembles of high- (emotion), and low-level (brightness) visual information, extracted from peripheral faces (presentation/encoding:200 ms), unintentionally influences perception of a central target face stimulus in individuals typically developing (TD) and with autism spectrum condition (ASC). Graded alterations in the summary intensities of the emotion and brightness of the peripheral stimuli modulated the perceptions of the target face in both TD and ASC. In contrast, when we measured goal-directed (overt) ensemble face- emotion and brightness perception, we found that in half of ASC the overt ensemble emotion perception was impaired than TD. Additionally, we repeated both experiments with a backward visual mask to restrict not just encoding but also background processing in the visual system to 200 ms. This revealed that the effect of peripheral ensembles on centre perception was present only with brightness at least in TD but of overt ensembles was evident with both emotion and brightness in TD and ASC alike. These results suggest that while ensemble statistics of low-level information derived automatically and rapidly (200 ms) from contextualized faces are used for target face perception, the same takes longer with high-level information. However, overt facial ensembles are rapidly processed in both TD and ASC.

## Introduction

Humans can efficiently extract summary statistics (e.g. mean, variance, range) from groups of visual entities, also known as ‘ensemble representation’^1^. This summarizing has not only been reported across simple objects and low-level visual features, such as size^2,3^, orientation^4,5^, brightness^6^, and colour^7^, but also higher-level visual features, such as facial-identity^8,9^, gender^10^, attractiveness^11^, and emotion^10,12^. Forming ensemble representation across a plethora of visual information allows the brain to statistically summarize related and relevant information into meaningful percepts that can efficiently guide behaviour and is generally accepted as a ‘global representation’^1^. However, if and to what extent the ensemble (global) representation computed from parts of an image influences the perception of a given target (local visual target of interest) within that image has been less explored to date.

Some human studies have demonstrated that the above phenomenon operates with various low- to mid-level features and objects as visual stimuli. Memory of features of an object (e.g. colour) was reported to be influenced by spatial context^13^, while remembered size of individual circles was biased by the mean of other circles^14^. Likewise, spatial frequencies of gabor-patches were biased by the previously presented patches^15^. Influence on visual short-term memory of shape, orientation, and colour of objects was found by manipulating the respective features of the non-target object sets in a change detection task^16^. More recently, this phenomenon was also reported with high-level visual stimuli, such as faces which were perceived as more attractive in groups than in isolation^11,17,18^.

All the above-mentioned studies found the effect of contextual ensemble statistics on the perception of the visual target, mostly using the visual memory paradigms with relatively prolonged encoding time (≥500 ms). While most studies measured this effect with simple, low-mid level visual stimuli, those which used high-level face stimuli measured the effect on attractiveness. Notably, one study measured face-attractiveness, adaptation after-effects, and direct effect of peripheral ensembles^11^.

Since top-down attentional resources can be recruited on a timescale of ~ 300 ms or more^19^, it is possible that top-down influences contributed to a certain degree in the effects observed in the studies above. Furthermore, not only are objects processed differently from faces^20^, but perception of facial attractiveness has also been found to be independent from other social information, such as emotion^21^. Thus, whether the ensemble statistics of the periphery of a briefly presented target image may also serve as contextual information to influence the visual perception of the given target, in making a perceptual judgment of relatively complex faces, is not entirely clear. We expected that the ensemble statistics extracted rapidly within a visual snapshot during one fixation (usually the duration of a saccade: ≤300 ms) could also serve to automatically influence target visual perception. This is particularly pertinent in light of the evidence that goal-relevant, ensemble statistics can be derived extremely quickly, with ~50 ms of encoding^22^. It is also of interest to investigate this phenomenon in ASC since they are known to process human faces differentially^23,24^, with well-documented delays in the latency of N170 event-related potentials in response to faces^25^. Furthermore, while deficits have been found to occur, at least in low-level global summarizing abilities^26,27^, this has been contradicted^28^. Nonetheless, idiosyncrasies concerning ASCs’ visual processing/perception and the possible implications of such perceptual biases regarding differences in visually guided cognition have been suggested^29^ and atypical perception is included as a recent diagnostic criterion for ASC as per the Diagnostic and Statistical Manual of Mental Disorders (DSM)-V^30^.

Here, we asked – (a) whether visual information (high-level: emotion; low-level: brightness) conveyed by relatively complex ensembles such as human faces presented for a short duration (~200 ms, with little time to recruit top-down attentional resources while encoding stimuli) could directly influence the perception of a target face, automatically (without intention), in making a perceptual judgment; and (b) whether the findings in TD also extend to ASC participants.

**Figure 1:**
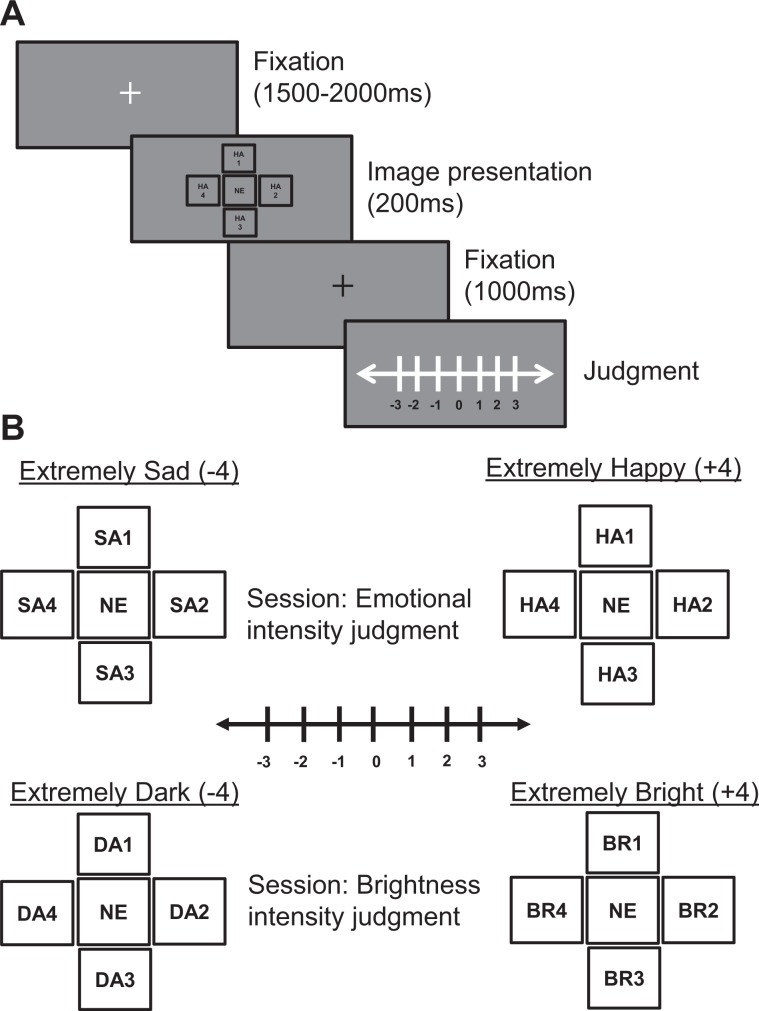
Experimental procedure. (**A**) A typical emotion-judgment trial. Each trial commenced with the presentation of a fixation cross in the centre of the screen (white fixation) that appeared for a random time duration (1,500–2,000 ms). The face image-set was then presented for 200 ms. Immediately afterwards, a black fixation cross appeared for 1,000 ms. This was followed by a key-pad response instruction. Participants were asked to accurately indicate a mentally computed representation using a seven-point Likert Scale (−3 to +3) without any time restriction. The trial ended with the key-press, and the next trial began after a blank screen of 500 ms. The face images were sourced from the Karolinska Directed Emotional Faces database (KDEF)^45^. Image types: Periphery (clockwise)- four Happy images (HA 1–4); Centre- neutral image (NE). Original images replaced by illustration for copyright reasons. (**B**) Experimental manipulation of the visual stimulus. (*Experiments 1 & 2*, emotion task-sessions; top) The peripheral neutral images (neutral emotion) were replaced by one additional happy image in increments of one at each level, until all four images were happy (extreme right; +4). The same process was applied for the opposite direction, this time using sad images (extreme left; −4). (*Experiments 1 & 2*, brightness task-conditions; bottom) Here, the peripheral neutral images (neutral brightness) were replaced by one additional bright or dark image in increments of one until the axis reached the maximum (extreme right; +4) or minimum (extreme left; −4), respectively. In each trial, the face image at the centre in each level was randomly selected to be happy/neutral/sad (emotion task-sessions; top) and bright/neutral/dark (brightness task-sessions; bottom). In *Experiment 1*, the effect of graded manipulation of the peripheral image set was tested on the perceived intensity of the centre face (emotion/brightness), whereas in *Experiment 2* the overall perceptual mean intensity of the entire five-image ensemble was tested. KDEF image types: top left [Extremely Sad], Periphery (clockwise)- four Sad images (SA 1–4); Centre- neutral image (NE); top right [Extremely Happy], Periphery (clockwise)- four Happy images (HA 1–4); Centre- neutral image (NE); bottom left [Extremely Dark] and bottom right [Extremely Bright], Periphery (clockwise)- four Dark images (DA 1–4); Centre- neutral image (NE); Image types: Periphery (clockwise)- four Bright images (BR 1–4); Centre- neutral image (NE). Original images replaced by illustration for copyright reasons.

To explore the above possibilities, we tested the performance of human participants (TD and ASC) in tasks concerning perceptual judgments of information from faces; this information pertained to both higher (emotion) and lower (brightness) levels of visual processing (Fig. 1). We presented face stimuli for 200 ms with little time for intentional coding (Fig. 1A). In Experiment 1, we tested whether the human visual system automatically depends on contextual ensemble summary statistics during judgments of a visual target (intensity of emotion/brightness of the centre face, Fig. 1B). We anticipated that summary intensity values of peripheral faces, conveyed as visual information, would affect the judgment of the target. Here, we measured the shift in participants’ responses regarding the visually perceived intensity of the target when the summary intensities of the peripheral stimuli were experimentally manipulated. In Experiment 2, we measured the same participants’ mean intensity perceptions of entire ensembles of faces (intended global ensemble perception) to test their goal-directed ability to integrate information sourced from the entire ensemble.

Thus, we parametrically manipulated the intensities of the stimulus ensembles in both experiments, as we examined the participants’ responses of global ensemble sensitization of local visual perception (covert visual-ensemble influence on overt visual perception; Experiment 1) as well as global ensemble visual perception *per se* (overt visual perception; Experiment 2), using both high-(emotion) and low-level (brightness) visual properties conveyed by human face stimuli. Additionally, by using a backward visual mask, we tested if the above perceptual effects (*Experiments 1 and 2*) were also a result of ensemble visual processing within 200 ms (not encoding duration of 200 ms) or it actually took longer for the process to gain effect (*Experiment 3*).

We found that irrelevant, contextual ensemble emotion and brightness information that were covertly presented, were effortlessly utilized both by TD and ASC, within a short presentation duration of 200 ms, to represent the visual target (the perceptual judgment of which was biased by the contextual ensemble statistics). Interestingly, the actual visual processing of the ensembles required >200 ms with emotion but not brightness information, at least in TD. In contrast, the overt ensembles of both emotion and brightness occurred within 200 ms in both TD and ASC. Here, a subset of ASC participants was impaired in emotion perception compared to TD.

## Results

### Effects of ensemble summary intensities of faces in the periphery (covert ensembles) on the perceptual intensity of the centre face (Experiment 1)

**Figure 2:**
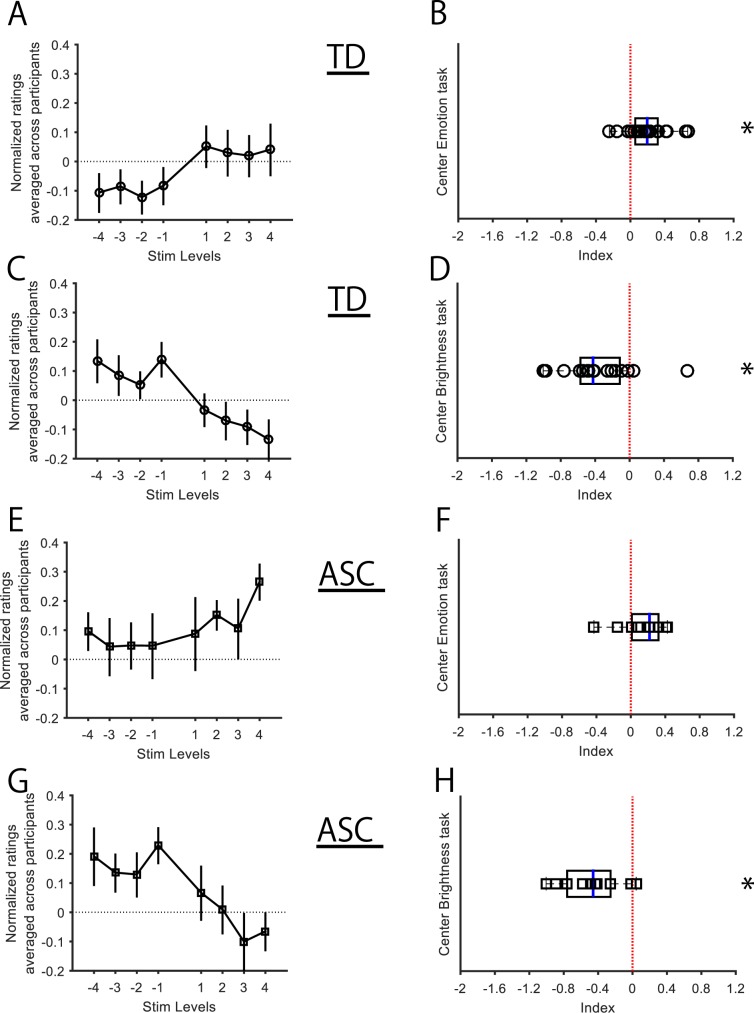
Effects of ensemble summary intensities of faces in the periphery (covert ensembles) on the perceptual intensity of the centre face (*Experiment 1*). (**A–D**) Data from TD (n = 19) are shown. Means ± SEMs of the raw judgment ratings across participants are shown for the positive (+1 to +4) and negative (−1 to −4) levels of stimulus manipulations relative to the zero level (black horizontal broken line) in the emotion (**A**) and brightness task-sessions (**C**), along with the distribution of their respective indices (black open circles; **B,D**). (**E–H**) Data from ASC (n = 10) are shown with the same conventions as TD: raw judgment ratings (emotion: **E**, brightness: **G**) and distribution of respective indices (black open squares; **F,H**). All statistical tests were conducted on the indices (**B,D,F,H**). *p < 0.05, Stim Levels (levels of experimental stimulus manipulation).

Manipulating the summary emotional intensities of the peripheral four-face ensembles demonstrated a trend of influencing the perceptual judgments of the emotional intensity pertaining to the central face (Fig. 2A). To quantitatively test the net change of perception as a function of the levels of stimulus manipulation, we calculated an index for each participant (see *Data Analysis*). The indices of the participants in this session were confirmed to follow a normal distribution (Lilliefors test; *p* = 0.5), and then one-sample *t*-test was conducted to test the difference of mean of the indices from the reference value zero. This returned a significant difference (*t*_(18)_ = 3.61; *p* = 0.002; Cohen’s *d* = 0.82; Fig. 2B), indicating that the indices’ mean of 0.20 (±0.05, standard error of mean (sem) was significantly greater than the reference value of zero and manipulating the peripheral ensemble emotion of the images had a direct influence on centre face perception.

To study the above effect in comparison with that of ensemble summary statistics pertaining to low-level visual properties of face images (i.e. brightness), we tested whether the perceptual judgment of the target (centre) face’s brightness shifted from zero when there was a graded change in the summary brightness values of the peripheral face ensembles. We consequently identified a clear effect in this regard across the TD indices (Fig. 2C). The normally distributed indices (Lilliefors test; *p* = 0.5), when entered into a one-sample *t*-test, revealed a marked effect of context (*t*_(18)_ = −4.06; *p* = 0.00073; Cohen’s *d* = 0.93; Fig. 2D), suggesting that the mean of −0.38 (±0.09, sem) was significantly different than zero, and manipulating the peripheral ensemble brightness of the images had an inverse influence on centre face brightness perception. There was neither any significant correlation between the emotion and brightness sessions (*p* = 0.31), nor did the indices (of either task) correlate with participants’ AQ scores (emotion: *r* = 0.21, *p* = 0.38; brightness: *r* = 0.15, *p* = 0.53).

The ASC participants’ target visual perception also demonstrated sensitivity towards the ensemble summary statistics of the contextual stimuli in both the higher (emotion; Fig. 2E,F) and lower levels of visual domains (brightness; Fig. 2G,H). However, separate one-sample *t*-tests (normality satisfied by Lilliefors test; emotion: *p* = 0.20; brightness: *p* = 0.50) revealed significant effects due to experimental manipulation of contextual brightness (mean = −0.48 ± 0.11; *t*_(9)_ = −4.37; *p* = 0.0018; Cohen’s *d* = 1.38; Fig. 2H) but not emotion (mean = 0.14 ± 0.08; *t*_(9)_ = 1.61; *p* = 0.14; Fig. 2F) on centre face perception. Please note that although there is an indication that the net perception of centre emotion was directly influenced by manipulation of the peripheral ensemble statistics, it is not significant because one of the participants showed extreme inverse perceptual modulation due to the experimental manipulation compared to the distribution of the rest of the participants (Fig. 2F). Even in this participant, the target visual perception was modulated nonetheless (see *Data analysis* for details), which is the actual goal of the experiment. Thus, testing whether the magnitude of perceptual modulation was different from zero, when we tested the absolute values of the indices by one sample *t* test, it revealed a significant effect of experimental manipulation in the emotion (mean = 0.25 ± 0.05; *t*_(9)_ = 5.51; *p* = 0.00037; Cohen’s *d* = 1.74; Fig. 2F) as well as brightness (mean = 0.49 ± 0.11; *t*_(9)_ = 4.64; *p* = 0.0012; Cohen’s *d* = 1.47; Fig. 2H) task-sessions.

There was no significant association between the emotion and brightness sessions (*p* = 0.66) or between the indices of either task session with participants’ AQ scores (emotion: *r *= 0.46, *p* = 0.18; brightness: *r* = 0.03, *p* = 0.94).

The results of this experiment suggest that summary intensity values computed subconsciously from contextual faces (in the periphery) elicit cognisable differences in visual perceptual judgments of the target face. Importantly, both TD and ASC participants demonstrated contextual sensitisation towards target face perception.

### Effects of ensemble summary intensities of all faces (overt ensembles) on the global mean perceptual intensity (Experiment 2)

**Figure 3:**
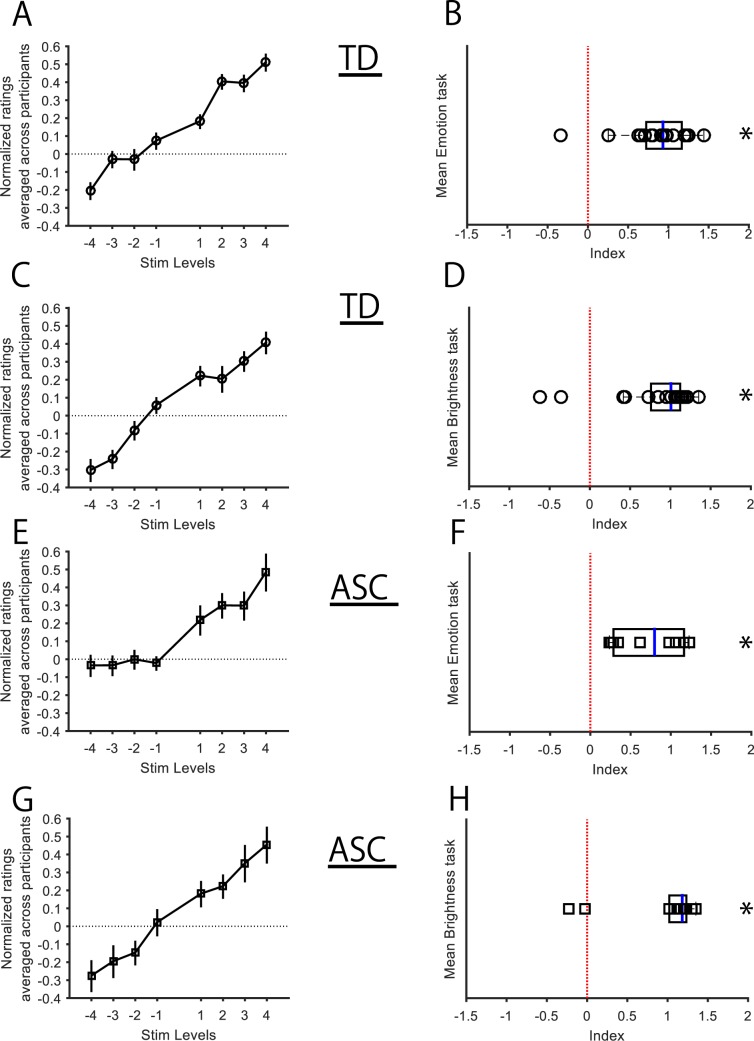
Effects of ensemble summary intensities of all faces (overt ensembles) on the global mean perceptual intensity (*Experiment 2*). (**A–D**) Data from TD (n = 19) are shown. Means ± SEMs of the raw judgment ratings across participants are shown for the positive (+1 to +4) and negative (−1 to −4) levels of stimulus manipulations relative to the zero level (black horizontal broken line) in the emotion (**A**) and brightness task-sessions (**C**), along with the distribution of their respective indices (black open circles; **B,D**). (**E–H**) Data from ASC (n = 10) are shown with the same conventions as TD: raw judgment ratings (emotion: **E**, brightness: **G**) and distribution of respective indices (black open squares; **F,H**). All statistical tests were conducted on the indices (**B,D,F,H**). *p < 0.05, Stim Levels (levels of experimental stimulus manipulation).

This experiment was similar in every respect to *Experiment 1*, with the exception that the participants were clearly instructed to utilise the visual information from all five images (the four images in the periphery and the centre image) to compute the global mean intensity of the entire image set. The intensity judgment scores of TD showed very clear modulation in response to relatively fine manipulations of the emotion (Fig. 3A,B) and brightness (Fig. 3C,D) of the face stimuli.

The TD indices for the emotion task session distributed normally (Lilliefors test: *p* = 0.13), and these indices’ mean of 0.87(±0.09, sem) was significantly greater than zero (one-sample *t-*test: *t*_(18)_ = 9.38; *p* = 2.36 × 10^−08^; Cohen’s *d* = 2.15; Fig. 3B). The distribution of the indices in the brightness task was non-normal (Lilliefors test of normality: *p* = 0.0012). Thus, a Wilcoxon signed rank test was conducted which demonstrated that the median for the brightness-task indices was 1.01 (0.36, inter-quartile range). This also significantly exceeded zero (*Z* = 3.62; *p* = 0.00029; Effect size = 0.83; Fig. 3D). Again, as in *Experiment 1*, neither did modulation of the perceptual judgments of the emotion task-session significantly correlate with brightness-session (*p* = 0.10) nor the tasks’ indices correlate with the participants’ AQ scores (emotion: *r* = 0.32, *p* = 0.17; brightness: *r* = −0.19, *p* = 0.43).

Similarly, in ASC, the emotion session indices were tested by one-sample *t* tests (Lilliefors *p* = 0.17) and the mean was found to be significantly larger than zero (mean = 0.73 ± 0.13; *t*_(9)_ = 5.44; *p* = 4.09 × 10^−04^; Cohen’s *d* = 1.72; Fig. 3E,F). The brightness session indices (Lilliefors *p* = 0.001; non-normal distribution) had a median of 1.18 (0.21, inter-quartile range), also exceeding zero (Wilcoxon signed rank test; *p* = 0.0098; Fig. 3G,H). As earlier, the indices of the two task-sessions did not demonstrate any significant association (*p* = 0.34) with AQ scores (emotion: *r* = 0.55, *p* = 0.09; brightness: *r* = 0.05, *p* = 0.88).

**Figure 4:**
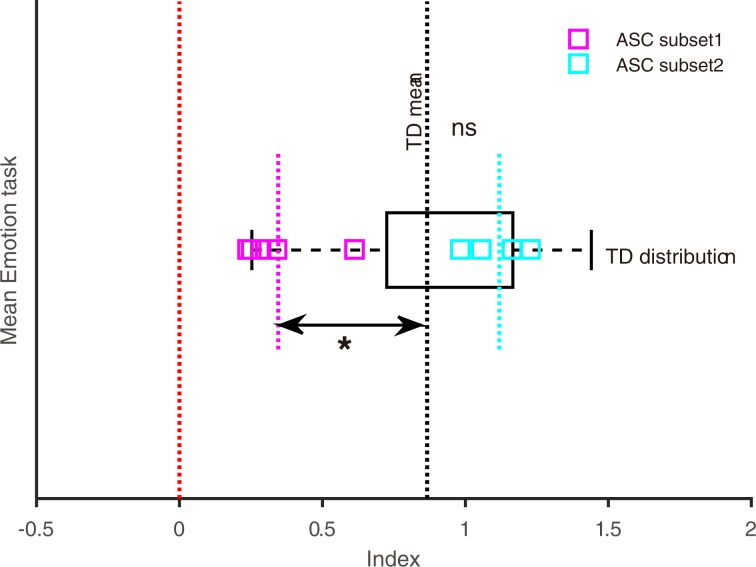
Subsets of ASC participants according to effects of overt ensemble emotion task of *Experiment 2*. The ASC indices (square markers) are plotted over the distribution of TD indices (black boxplot) in the emotion task-session. Note that the two subsets of ASC participants (five magenta and cyan squares each) fell on opposite sides of the mean of TD indices (black vertical broken line). *p < 0.05, significant difference between mean of indices in subset1 (magenta, vertical broken line) and TD mean. ns *p* > 0.05, insignificant difference between mean of indices in subset2 (cyan, vertical broken line) from TD mean. Red broken line indicates no effect of stimulus manipulation.

Notably, the ASC data from the emotion task-session when split by its median (0.79) indicated two distinct subsets of participants falling on either side of the TD mean (0.87, black broken line; Fig. 4). Five ASC performed poorly on this task (magenta squares, mean age = 31.60; mean AQ = 35.40; participant ID: 1, 7, 3, 6, 9; Table 1) whose performance (mean of indices = 0.34; <TD mean) was worse than the other five ASC (cyan squares, mean age = 23.40; mean AQ = 30.40) who performed much better (mean of indices = 1.11; >TD mean). The difference of means between each ASC subset (magenta and cyan broken vertical lines) and TD (black broken vertical line) was tested using permutation based independent t-test (20000 permutations) as previously described^31^. This approach is suggested to be better when sample size is less per group^32^. While the mean of ASC subset 1 (magenta broken vertical line) was significantly lower (t = −2.79; *p* = 0.0094), the ASC subset 2 (cyan broken vertical line) did not differ from TD (t = 1.37; *p* = 0.18). The difference between ASC subset 1 and TD was significant even at the Bonferroni corrected threshold *p*-value (0.05/2 = 0.025).

**Table 1:** Demographics of ASC participants. ADHD: Attention Deficit Hyperactivity Disorder; ASD: Autism Spectrum Disorder; LD: Learning Disorder; PDD: Pervasive Developmental Disorder.

ID	Age (yrs)	Gender (M/F)	AQ score	Diagnosis/medications (pharmacological category)
ASC-1	20	M	35	high AQ/none
ASC-2	32	M	34	high AQ/none
ASC-3	37	F	31	clinically diagnosed PDD, ADHD, LD/Duloxetine (selective serotonin and norepinephrine reuptake inhibitor), Zopiclone (non-benzodiazepine-hypnotic)
ASC-4	23	M	32	clinically diagnosed PDD/none
ASC-5	25	M	24	clinically diagnosed PDD/none
ASC-6	45	M	34	clinically diagnosed ASD/Quetiapine (selective monoaminergic antagonist-antipsychotic), Clonazepam (benzodiazepine-anticonvulsant)
ASC-7	35	M	41	clinically diagnosed ASD and ADHD/Methylphenidate (norepinephrine and dopamine reuptake blocker-central nervous system stimulant)
ASC-8	22	M	29	clinically diagnosed PDD/none
ASC-9	21	M	36	clinically diagnosed PDD/Atomoxetine (non-stimulant, selective norepinephrine reuptake inhibitor)
ASC-10	15	M	33	clinically diagnosed ASD and ADHD/Methylphenidate(norepinephrine and dopamine reuptake blocker-central nervous system stimulant), Modafinil

These results suggest that in response to overt task demands, TDs can generally efficiently integrate visual information from face ensembles whereas a subset of ASC has likely a deficit in this ability, particularly in summarizing ‘emotion’ information.

### Effects of covert and overt ensembles with backward visual masking (Experiment 3)

The above effects observed in *Experiments 1 and 2* occurred with a stimulus (face ensembles) presentation of 200 ms for encoding but the actual processing of the stimulus could have also happened in the visual system on a later timescale in the intervening blank of 1,000 between the stimulus offset and judgment onset (Fig. 1A). To ascertain if the perceptual effects in *Experiments 1 and 2* were actually a result of visual processing of the stimulus within 200 ms, we modified the event timeline of the task by including a high-contrast backward visual mask for 1,000 ms immediately following the stimulus offset (Supplementary Information Fig. 1) to suppress processing of the stimulus in the visual system. The data collected were tested by permutation-based one-sample t-tests (all possible permutations) for difference of the mean of indices from the reference value of zero. This task manipulation in TD revealed that peripheral ensemble emotion information (covert ensembles) had no perceptual effect on centre emotion perception (mean ± sem of indices = 0.025 ± 0.09; *t*_(10)_ = 0.28; *p* = 0.79; Supplementary Information Fig. 2B), whereas the perceptual effect of peripheral brightness information on centre brightness was significant (mean ± sem of indices = −0.55 ± 0.13; *t*_(10)_ = −4.36; *p* = 0.0039; Cohen’s *d* = 1.31; Supplementary Information Fig. 2D). The same manipulation produced no significant perceptual effects either with emotion (mean ± sem of indices = −0.15 ± 0.15; *t*_(5)_ = −0.98; *p* = 0.38; Supplementary Information Fig. 2F) or brightness (mean ± sem of indices = −0.24 ± 0.22; *t*_(5)_ = −1.13; *p* = 0.28; Supplementary Information Fig. 2H) in ASC. However, it is possible that due to the relatively smaller sample size we could not detect an effect here.

By contrast, the perceptual effects of goal-directed, overall ensemble of faces (overt ensembles) were significant with emotion (mean ± sem of indices = 0.69 ± 0.14; *t*_(9)_ = 4.99; *p* = 0.002; Cohen’s *d* = 1.58; Supplementary Information Fig. 2B) and brightness (mean ± sem of indices = 0.93 ± 0.22; *t*_(9)_ = 4.16; *p* = 0.0059; Cohen’s *d* = 1.31; Supplementary Information Fig. 2D) in TD as well as with emotion in ASC (mean ± sem of indices = 0.83 ± 0.19; *t*_(5)_ = 4.39; *p* = 0.031; Cohen’s *d* = 1.79; Supplementary Information Fig. 2F). The indices of brightness task failed to attain significance by a narrow margin (mean ± sem of indices = 0.82 ± 0.20; *t*_(5)_ = 3.98; *p* = 0.062; Cohen’s *d* = 1.62), but this could be due to one outlier showing opposite effect than the rest of the small sample of ASC (Supplementary Information Fig. 2H).

## Discussion

In the first experiment, we found that percepts of high- and low-level (sensory) target visual information are sensitive to the summary statistics provided by the respective ensemble properties of contextual stimuli. Participants determined the summary intensities of the contextual face ensembles and encoded the target face intensities relative to those of the summary intensities in such a way that manipulating the context produced a systematic modulation of the perceptual responses in both TD and ASC.

We tested the effect that manipulating peripheral, global summary measures (of the context stimuli) has on the perception of local features of stimuli by parametrically varying the ensemble information while encoding. It is worth noting here that this effect was quantitatively evident when the contextual stimuli were behaviourally irrelevant (had no task relevance; participants were told to ignore them) and the stimuli (both context and target) were co-presented for a brief period (200 ms). Thus, despite the task demand of perceiving only the intensity of the centre face, the participants’ visual systems subconsciously accounted for the summary values provided by the peripheral stimuli. The influence of manipulating the global summary measures of the peripheral faces on centre face perception was mutually in opposite directions for the emotion and brightness task sessions in both groups. This indicates that potentially different neural mechanisms were at play in producing the target perceptual bias due to ensemble statistics extracted covertly from high- and low-level visual information from faces. The stimuli were presented for 200 ms, which is approximately the average rate at which humans make a saccade (~200–300 ms)^33^. Therefore, it is unlikely that participants could have sampled the face information from all of the images in a trial, particularly when the task instruction was clearly focussed on the target-centre image. That we nevertheless observed an effect of peripheral modulation is possibly because the human visual system is capable of extracting summary, visual-ensemble statistics without serially foveating on each image, which also corroborates a recent finding^34^. Although earlier studies have reported rapid extraction of task-relevant ensemble percepts in humans within time scales of 50–500 ms^10,22^, here we report distinct encoding of visual targets relative to the extracted summary values of their task-irrelevant ensemble (context) within 200 ms, which ultimately influenced the visual perception of the relevant target.

There are previous accounts of superior local, visual processing and attention in ASC^29,35^. In a visual acuity task, the spatial gradients of visual attention (to cued locations) fell more sharply between 2.46–4.51° of the visual angle in ASC than TD, when a target–distractor pair were shown for 67 ms^35^. Since our *Experiment 1* required task-based deployment of visual attention (for 200 ms) only to the centre–face image (~4 × 4°), we also expected the effect of peripheral images (~5° apart in four directions, from the mid-point of the target) to be minimal in ASC. However, we found TD and ASC sensitization to similar extents. The result suggests that task-irrelevant visual information from surrounding faces (presented within relatively comparable spatial extents but over a greater time span than previously reported^35^, may still modulate target visual perception, revealing the influence ensemble summary measures of a visual context can exert on ASC local visual perception.

Visual context has been referred to as the ‘glue’ that integrates different elements into a coherent percept; it can influence our eye-movements and attention, thereby guiding perception^36^. In accordance with this, a ‘relevant background’ may also be interpreted as visual context. Recent reports have demonstrated that the visual system can automatically utilise contextual frames of reference (‘background coordinates’) to control saccades in less than 150 ms^37^, and skilled arm movements within 300 ms^38^, towards visuospatial targets. Further, the role of voluntary top-down attention regarding neuronal responses at different stages of information processing in the visual cortex is well-known^39^. The engagement of top-down attention generally allows one to prioritise goal-relevant processing of stimulus information; in the present study, this priority should have been placed on the centre face, which would have attenuated the effect of the context on visual perception of the centre face. However, since our encoding stimuli were presented only briefly (for 200 ms), top-down attention may not fully explain our results, as studies have reported that a timescale of ~300 ms or more is required for the engagement of top-down attention^19^. Therefore, we consider that our findings regarding encoding the target relative to background ensemble summary statistics were also the result of automatic, exogenous attentional processes^19,40^. It is noteworthy however, that as revealed by our Experiment 3, the peripheral ensembles presented for 200 ms (encoding duration) actually take >200 ms for visual processing and thereby for the perceptual modulation to gain effect at least with high-level emotion information.

A recent study has demonstrated that, in human participants, contextual stimuli have a marked influence on the detection of changes in facial expressions^41^. Using affective priming and a bi-directional response task, the authors reported that when participants were exposed to an intervening context (presented for 1,000 ms), they returned a greater proportion of correct judgments of the direction of change between pre- and post-context facial expressions. This applied to trials where valence was consistent with the actual direction of change in the facial expressions (either positive or negative). Although some aspects of the overall findings of this study are similar to those of the present study, our study has some salient differences. The above study used a ‘priming’ paradigm, which essentially preceded the actual tasked stimulus, and focussed on subtle changes in facial emotions^42^. The duration of the priming was 1,000 ms, and the latency between presentations of the priming and target was 1,250 ms; both durations were possibly intended to facilitate top-down conditioning of the target evaluation based on affective memory of the prime. On the contrary, our task involved concurrent representation of the target and extraction of summary contextual valence, with little room for encoding by conscious intention (200 ms). This information pertaining to both visual domains in ASC and the high-level domain in TD was visually processed on time scales of 200–1000 ms, which may have later led to the perceptual bias in our results. Consequently, we speculate that a similar but still distinct mechanism might be involved in our study, and feel that our results supplement the previous study’s findings^41^.

It has also been reported that perceptual judgments of facial emotional intensity in humans is susceptible to implicit racial biases^43^. Further, a recent study has demonstrated that the facial features that one learns over time influences how faces are evaluated socially^44^. Since our entire participant pool comprised of Japanese adults, who were likely to have been relatively unfamiliar with Caucasian faces, whether our experimental results, which were obtained using stimuli comprising Caucasian faces^45^, would differ if Asian or specifically Japanese facial stimuli were used merits future exploration.

In *Experiment 2*, we measured whether participants’ performances could be flexibly switched to an entirely global ensemble perception when they were given an instruction to mentally compute the mean visual percept (as a summary measure) of the two visual properties (emotion, brightness) based on all five face images. The purpose was to test how participants consciously, visually averaged global scenes, rather than the sensitization of their local perception by summary measures of the covert global context (tested in *Experiment 1*). Consequently, participants (TD and ASC) overall demonstrated robust effects regarding averaging both types of visual property (emotion and brightness) in response to the manipulations of the stimuli. In contrast to *Experiment 1*, however, we found that the perceptual shift as a result of overt global summarizing from the face ensembles were in a similar direction with both high- and low-level visual information (emotion and brightness). Therefore, our results support previous reports that the visual system is adept at summarising ensemble information within a short presentation span^1^. In fact, we observed a better modulation of perceptual judgment in this task than for *Experiment 1*. This may have been possible due to the availability of a clear summarising strategy: the task instruction to consciously compute the mean emotion and brightness of the entire ensemble. Engaging attention while encoding has been proposed to result in more reliable representations^1^ which may have led to greater effect sizes. On the other hand, in *Experiment 1*, the effect of summary measures of the peripheral (contextual) ensemble was measured indirectly/covertly, with the participants only consciously judging the centre image. Moreover, different summarising strategies (mean/variance/range) could have been adopted in *Experiment 1* (both across trials for individual participants and across different participants), which collectively might have led to a relatively smaller effect size here. However, 50% of the ASC participants demonstrated an impaired ability to summarize emotion information, in particular. Efficiency of goal-directed global visual processing in ASC has yielded contradictory results in the past and, as mentioned above, we think this requires better understanding^29^. Finally, the perceptual effects related to information from low- and high-level visual domains in *Experiment 2* were evident in both TD and ASC with a shorter visual processing duration of 200 ms, as revealed by our *Experiment 3*. This could have been due to the attentional benefits afforded by the clear task instruction at the encoding step.

It is pertinent to note here that the size of our ASC participant pool was rather modest and its composition heterogeneous. Replication of our findings in a larger and more homogenous ASC participant pool than ours would be required to unequivocally confirm these effects in ASC.

Taken together, our results demonstrate that in the brief presence (200 ms) of contextual visual information from faces, a target face could be interpreted with respect to the ensemble summary statistics computed automatically from the irrelevant context, so as to influence a visual perceptual judgment in both TD and ASC. While the actual visual processing in this phenomenon takes longer (>200 ms) with high-level emotion information in both TD and ASC, this processing can happen rapidly (≤200 ms) with low-level brightness information, at least in TD. Goal-directed overt ensemble summary statistics however, can be computed rapidly in both TD and ASC regardless of the type of visual information.

## Methods

### Participants

We recruited 29 participants for this research (comprising TD and ASC). All had normal or corrected to normal vision, and provided written informed consent to participate in two experiments. Each participant performed the two experiments on different days (for all participants, the gap between the two experiments was >24 hours except for one ASC participant, ASC-10, for whom the gap was three hours). All participants answered the Japanese version of the autism spectrum quotient (AQ) test, which measures autistic traits. The study design was approved by the Ethics Review Board of the National Rehabilitation Centre for Persons with Disabilities, Tokorozawa (Saitama), Japan and all experiments were carried out in accordance with the guidelines for human participants laid down by the Ethics Review Board. All participants were remunerated for participating in the research. The face images were sourced from the Karolinska Directed Emotional Faces database (KDEF)^45^, which is permitted to use for non-commercial scientific research purposes.

#### Typically developing individuals

Nineteen TDs participated in both *Experiment 1 and 2* (mean age 22.09 years; age range 18–26 years; 12 women). All returned AQ scores ≤30 (mean AQ score = 17.16). None reported a family history of ASC and they were free from any history of neurological disorders.

#### Individuals with ASC

Ten ASCs (mean age 27.50 years; age range 15–45 years, one woman) participated in both *Experiments 1 and 2*. Eight were clinically diagnosed cases of autism spectrum disorders and/or pervasive developmental disorders. Three other participants who returned a high AQ score (criterion of ≥33) were also classified as ASC^46^. Six of the ASC participants were on medications (see Table 1 for details).

### Apparatus and general task procedures

For the experiments, participants were seated with their arms resting on a desk in front of them, and with their heads stabilised using a chin rest and head rest. The stimuli were presented on a 23-inch colour monitor (1,920 × 1,080 pixels, 60 Hz; DELL, Round Rock, TX) placed at a distance of 57 cm from the participants, with the edges subtending visual angles of 50.5° × 28.4°. The experiment was controlled using the Psychophysics Toolbox (Brainard, 1997), which was operated using MATLAB 2015a (Mathworks, Natick, Massachusetts, USA).

At the beginning of each trial, the participants were instructed to fixate on a white cross in the centre of the screen (0.5° × 0.5°), which was presented for a randomly determined duration of between 1,500 and 2,000 ms (Fig. 1A). The offset of the cross was followed without delay by the presentation of a set of five face images; all images were presented with the edges of the image subtending ~4 × 4°. The image set was positioned such that the central face image was directly at the centre of the screen. The four flanking face images were positioned in four perpendicular directions (right and left; top and bottom) from the centre image. The respective centres of the four flanking images were 5° from the mid-point of the centre image with a gap of 1° among the edges of the images. The entire image set was presented for 200 ms. Subsequently, a black fixation cross appeared at the centre of the screen for 1,000 ms. This was followed by the presentation of a seven-point Likert scale (Likert, 1932), through which participants were asked to report their perceived intensities of either the emotion or brightness (see below) of the centre face. They answered using a response pad (the seven-key RB-740 pad, Cedrus Corporation, San Pedro, CA), and there was no time limit for providing responses. The trial ended as soon as the participant made a keypad response and, after a blank screen for 500 ms, the next trial began. The face images were sourced from the Karolinska Directed Emotional Faces database (KDEF)^45^. For the emotion-judgment tasks, faces expressing happy, neutral, and sad emotions were selected. For each emotion, we selected 35 female and 35 male images, meaning the overall set for the emotion task comprised 70 × 3 = 210 images. First, the images were converted to grayscale by setting the hue and saturation to zero, while preserving the luminance. Then, the brightness of each image was adjusted to the same level (mean = 128, in a range of 0–255). The images were then used for the emotion-judgment tasks in both experiments. For the brightness-judgment tasks, the grayscale-converted images that expressed only neutral emotions (total 70 images: 35 female +35 male faces) were used. The brightness of these images was then modified, being both increased by 30% (brighter) and decreased by 30% (dimmer) of the original value (neutral), to form three sets: bright, neutral, and dark types.

The image properties were managed using the Image Processing Toolbox ver. 9.2 of MATLAB 2015a (MathWorks, Natick, Massachusetts, USA). All participants performed 5–10 practice trials before commencing each experiment.

#### Experiment 1

Participants were presented with sets of five face images. The image at the centre of the screen was surrounded by four others (peripheral images), as described above. During the trials, the summary intensity value of the four-image ensemble of peripheral images was manipulated in a graded manner using nine assigned stimulus levels (ranging from a maximum of +4 (extremely happy/bright) to a minimum of −4 (extremely sad/dark); Fig. 1B). Participants were instructed to only consider the centre image (visual target). They were asked to carefully form a mental representation of the relative intensities of emotion (one experiment session) and brightness (in another experiment session) of the central face and to indicate their judgments using a seven-point Likert Scale (−3 (extremely sad/extremely dark) to +3 (extremely happy/extremely bright); Fig. 1B). They were directed to ignore the four peripheral face images that would be presented concurrently with the target image. All participants performed the two sessions (emotion and brightness), each comprising 81 trials, with intermittent rest periods. Each individual stimulus level (from −4 to +4) comprised nine trials (3 trials each with centre - happy/neutral/sad or bright/neutral/dark). Thus, there were 9 stimulus levels × 9 trials/stimulus level = 81 trials.

Since the task instruction was to focus only on the briefly presented visual target, we expected that the centre image would capture the maximum attention. Nonetheless, if the visual target was perceived relative to the ensemble of the four peripheral images, the judged intensity of the visual target would shift systematically with the respective graded alterations in the summary intensity values of the ensemble.

#### Experiment 2

Similar to Experiment 1, participants were presented with sets of five face images, but on this occasion they were instructed to view all five face images and to mentally compute their mean emotional or brightness intensity. The peripheral images were used to manipulate the mean intensity of the entire five-image ensemble in a graded manner, based on nine assigned stimulus levels as before (ranging from a maximum of +4 (extremely happy/bright) to a minimum of −4 (extremely sad/dark; Fig. 1B). Participants were asked to carefully form a mental representation and to then indicate their judgments of the average intensities of the emotion and brightness of the faces (two separate sessions) using the same seven-point Likert Scale as in Experiment 1. Each participant performed 81 trials in the two separate sessions, with an intermittent rest period. As before, each individual stimulus level (from −4 to +4) comprised nine trials (3 trials each with centre - happy/neutral/sad or bright/neutral/dark): 9 stimulus levels × 9 trials/stimulus level = 81 trials.

There was a clear task instruction to average the global ensemble, and we expected that if the participants could fulfil the task demands, there would be a systematic modulation of the judged average intensities as the overall means of the five image ensembles were manipulated from low to high, as previously reported^6,9^.

The order of measuring the participants’ responses for experiments 1 and 2, as well as between sessions 1 (emotion-judgment task) and 2 (brightness-judgment task) of both experiments was counterbalanced across the participants.

#### Experiment 3

This was done with a smaller sample of TD (N = 11; mean age 24.80 years; age range 20–34 years; six women) and ASC (N = 6; mean age 21.67 years; age range 16–26 years; no women; Table 1: ASC # 2,4,5,8,9,10) participants. Here, the participants performed a modified version of both *Experiment 1* and *Experiment 2* on the same day with an intermittent rest period of ~15 minutes. Specifically, a high-contrast backward visual mask (perlin noise) was presented (duration 1,000 ms) immediately after the stimulus (face ensembles) offset (Supplementary Information Fig. 1) in order to restrict the visual processing of stimuli to 200 ms (same as the presentation/encoding duration). Other task events were same as Fig. 4A. All other aspects of task instruction and execution were the same as described above for *Experiment 1* and *Experiment 2* respectively.

#### Data analysis

Custom codes were written to analyse the data obtained through the keypad responses. The following is an explanation of the calculation steps for a single participant. Initially, the means of the nine responses for each stimulus level (−4 to +4) were calculated. These individual response values for each stimulus level were then re-scaled with respect to the maximum and minimum scores (across all stimulus levels between −4 and +4) to obtain normalised scores on an interval scale [0–1]. Next, the response values corresponding to the ‘zero stimulus level’ (image set in which emotion/brightness types of all four peripheral images were neutral, i.e. either all neutral emotions/all neutral brightness) were subtracted from the values of each stimulus level: +1 to +4 (positive levels) and −4 to −1 (negative levels). Finally, a simple index was calculated by dividing the difference of mean responses (across positive and negative stimulus levels respectively) by the mean taken across the original (un-subtracted) responses of all the positive and negative stimulus levels (Eq. 1). This index was used to evaluate the overall modulation of the judged perceptual intensities as a result of the manipulation of the stimulus levels.

1 $$Index=\frac{(\frac{1}{n}\times \mathop{\sum }\limits_{i=1}^{n}Pn-\frac{1}{n}\times \mathop{\sum }\limits_{i=1}^{n}Nn)}{\frac{1}{N}\times (\mathop{\sum }\limits_{i=1}^{n}Pn+\mathop{\sum }\limits_{i=1}^{n}Nn)}$$

where,

*P*_*n*_ = normalised responses of participants for ‘positive stimulus level’ trials

*N*_*n*_ = normalised responses of participants for ‘negative stimulus level’ trials

*N* = total number of positive and negative stimulus levels (eight)

Through this process, two indices were calculated for the two separate sessions (emotion- and brightness-judgment tasks) of all the three experiments. Such indices were calculated for all participants and used to test the difference of these indices from zero. If there was an effect of the experimental manipulation on the visual perceptual judgments, the mean value of indices would significantly be far away from (different from) zero. The sign of the indices (positive or negative) would indicate the direction of overall perception as a result of the experimental manipulation. Thus, in *Experiment 1*, indices greater than zero (positive indices) would suggest that manipulation of peripheral, ensemble intensities (face emotion along the sad-happy axis/face brightness along the dark-bright axis) directly influenced centre face perception, i.e. increased ensemble intensities of peripheral happiness/brightness directly led to increased perceptual intensities of happiness/brightness of the centre face. By comparison, indices less than zero (negative indices) would point at an inverse influence of the manipulation of peripheral, ensemble face emotion/brightness on centre face perception, i.e. increased ensemble intensities of peripheral happiness/brightness led to decreased perceptual intensities of happiness/brightness respectively of the centre face. Similarly, in *Experiment 2*, positive indices would indicate the direct effect of manipulation of the ensemble intensities (emotion/brightness) of the entire face image-set whereas negative indices would indicate the inverse effect of the same manipulation on global mean ensemble perception.

All statistical analyses were conducted using the Statistics and Machine Learning Toolbox (version 10.0) of MATLAB 2015a (MathWorks, Natick, Massachusetts, USA). For all purposes, *p* < 0.05 was considered to indicate significance.

## Data and code availabilty

The datasets and codes related to the published results are available from the corresponding author upon reasonable request.

## Supplementary information

Supplementary Information.
