# Supplementary material for: Perceptual effects of fast and automatic visual ensemble statistics from faces in individuals with typical development and autism spectrum conditions
Source: Sci Rep. 2020 Feb 7;10:2169. doi: 10.1038/s41598-020-58971-y (PMC7005810; doi:10.1038/s41598-020-58971-y)
Supplement: Supplementary file 1 — Supplementary Information. [file 41598_2020_58971_MOESM1_ESM.docx]

**Suppementary Information**

Perceptual effects of fast and automatic visual ensemble statistics from faces in individuals with typical development and autism spectrum conditions

Mrinmoy Chakrabarty ^1^, Makoto Wada ^1^.

^1^ Developmental Disorders Section, Department of Rehabilitation for Brain Functions, Research Institute of National Rehabilitation Center for Persons with Disabilities, Saitama 359-8555, Japan

**Corresponding Author:** Makoto Wada M.D., Ph.D.

Chief, Developmental Disorders Section, Department of Rehabilitation for Brain Functions, Research Institute of National Rehabilitation Center for Persons with Disabilities, 4-1, Namiki, Tokorozawa, Saitama, 359-8555, JAPAN.

TEL: +81-4-2995-3100 (ex. 2578); FAX: +81-4-2995-3132; E-mail: [wada-makoto@rehab.go.jp](mailto:wada-makoto@rehab.go.jp)

**Supplementary Figure 1**

**Supplementary Figure 2**


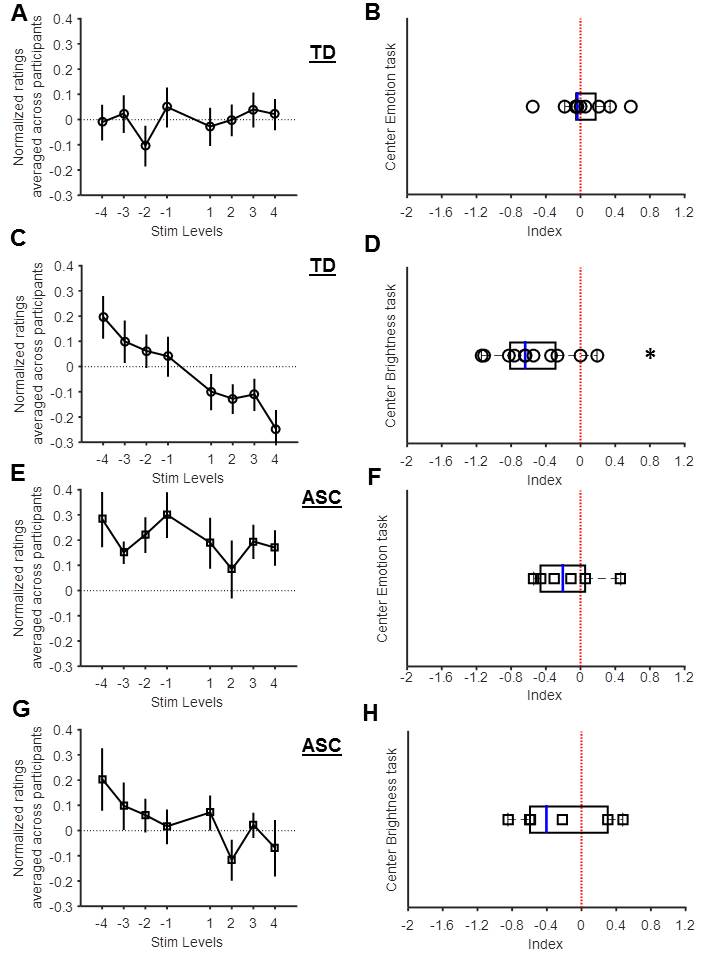


**Supplementary Figure 3**

**
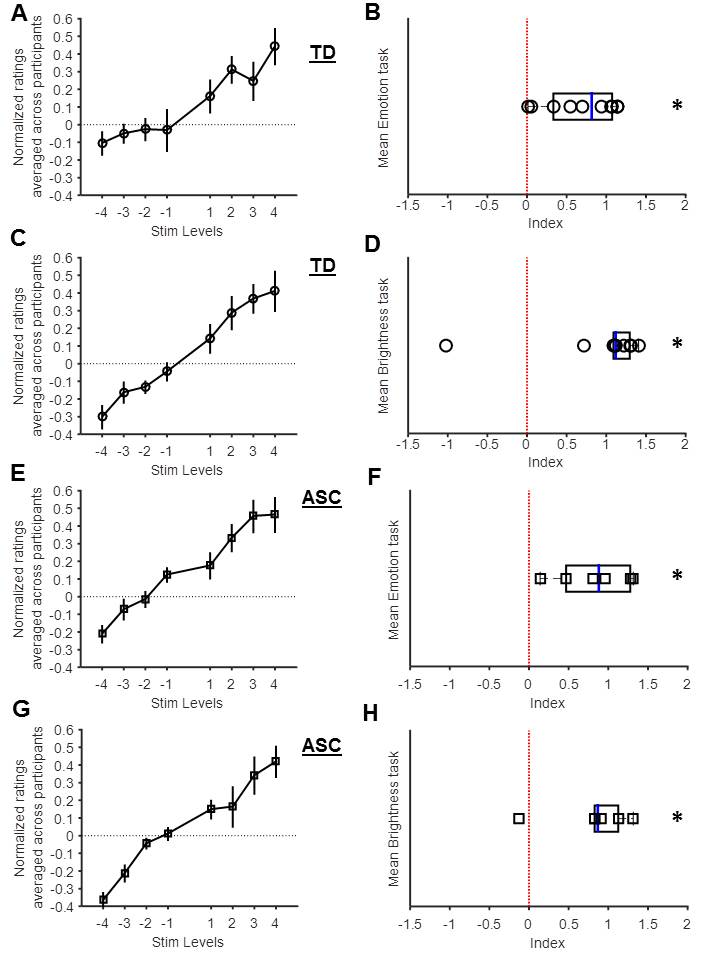
**

**Supplementary Figure Legends**

**Supplementary Figure 1. Experimental procedure** A typical emotion-judgment trial. Each trial commenced with the presentation of a fixation cross in the centre of the screen (white fixation) that appeared for a random time duration (1,500–2,000 ms). The face image-set was then presented for 200 ms which was followed without delay by a high-contrast visual mask (perlin noise) with a central black fixation cross for 1,000 ms. This was followed by a key-pad response instruction. Participants were asked to accurately indicate a mentally computed representation without any time restriction. The trial ended with the key-press, and the next trial began after a blank screen of 500 ms. All other aspects were same as described in **Figure 1B** and ***Apparatus and general task procedures***. The face images were sourced from the Karolinska Directed Emotional Faces database (KDEF) ^1^. KDEF image IDs:Periphery (clockwise)- Periphery (clockwise)- four Happy images (HA 1-4); Centre- neutral image (NE). Original images replaced by illustration for copyright reasons.

**Supplementary Figure 2. Effects of covert ensembles with backward visual masking.** (A - D) Data from TD (n=11) are shown. Means ± SEMs of the raw judgment ratings across participants are shown for the positive (+1 to +4) and negative (-1 to -4) levels of stimulus manipulations relative to the zero level (black horizontal broken line) in the emotion (A) and brightness task-sessions (C), along with the distribution of their respective indices (black open circles; B, D). (E - H) Data from ASC (n=6) are shown with the same conventions as TD: raw judgment ratings (emotion: E, brightness: G) and distribution of respective indices (black open squares; F, H). All statistical tests were conducted on the indices (B,D,F,G). * *p* < 0.05, Stim Levels (levels of experimental stimulus manipulation).

**Supplementary Figure 3. Effects of overt ensembles with backward visual masking.** (A - D) Data from TD (n=10) are shown. Means ± SEMs of the raw judgment ratings across participants are shown for the positive (+1 to +4) and negative (-1 to -4) levels of stimulus manipulations relative to the zero level (black horizontal broken line) in the emotion (A) and brightness task-sessions (C), along with the distribution of their respective indices (black open circles; B, D). (E - H) Data from ASC (n=6) are shown with the same conventions as TD: raw judgment ratings (emotion: E, brightness: G) and distribution of respective indices (black open squares; F, H). All statistical tests were conducted on the indices (B,D,F,G). * *p* < 0.05, Stim Levels (levels of experimental stimulus manipulation).

**References**

1. Flykt, A., Lundqvist, D., Flykt, A. & Öhman, A. The Karolinska directed emotional faces (KDEF). *CD ROM from Dep. Clin. Neurosci. Psychol. Sect. Karolinska Institutet* (1998). doi:10.1017/S0048577299971664
